# Risk of rapid progression to dialysis in patients with type 2 diabetes mellitus with and without diabetes-related complications at diagnosis

**DOI:** 10.1038/s41598-023-43513-z

**Published:** 2023-09-29

**Authors:** Hong-Mo Shih, Wen-Chen Tsai, Pei-Yu Wu, Li-Ting Chiu, Pei-Tseng Kung

**Affiliations:** 1 Department of Public Health, China Medical University, Taichung, Taiwan; 2 Department of Emergency Medicine, China Medical University Hospital, Taichung, Taiwan; 3 School of Medicine, College of Medicine, China Medical University, Taichung, Taiwan; 4 Department of Health Services Administration, China Medical University, Taichung, Taiwan; 5 Department of Psychology, Asia University, Taichung, Taiwan; 6 Department of Medical Research, China Medical University Hospital, China Medical University, Taichung, Taiwan; 7 Department of Healthcare Administration, Asia University, 500, Lioufeng Rd., Wufeng, Taichung, 41354 Taiwan

**Keywords:** Diseases, Endocrinology, Health care

## Abstract

Many adults with diabetes mellitus are unaware worldwide. The study objectives aimed to evaluate the risk of dialysis within 5 years of diagnosis between patients with newly diagnosed diabetes with and without diabetes-related complications. A retrospective longitudinal nationwide cohort study was conducted. Patients diagnosed with diabetes between 2005 and 2013 were followed up until 2018. They were categorized based on the presence or absence of complications, the number of complications, and the diabetes complications severity index (DCSI) scores. Dialysis outcomes were determined through the Registry of Catastrophic Illness from the National Health Insurance Research Database. Among the analyzed patients, 25.38% had complications at diagnosis. Patients with complications at diagnosis had a significantly higher risk of dialysis within 5 years (adjusted hazard ratio: 9.55, 95% confidence interval CI 9.02–10.11). Increasing DCSI scores and the number of complications were associated with higher dialysis risks. Patients with one complication had a 7.26-times higher risk (95% CI 6.83–7.71), while those with ≥ 3 complications had a 36.12-times higher risk (95% CI 32.28–40.41). In conclusion, newly diagnosed diabetes patients with complications face an increased risk of dialysis within 5 years. The severity and number of complications are directly linked to the risk of dialysis within this timeframe.

## Introduction

The global prevalence of diabetes mellitus (DM) among adults aged 20–79 years was approximately 9.3% in 2019^1^. Furthermore, according to an estimation from the International Diabetes Federation, half of all adults aged 20–79 years with DM are unaware that they have this condition^1^. In Taiwan, 11.46% of adults reportedly have DM^2^. DM-related medical expenses account for approximately 4.66% of all medical expenses, making DM the third-highest generator of medical expenses in Taiwan^3,4^.

Diabetic kidney disease (DKD) and diabetic nephropathy are the leading causes of end-stage renal disease (ESRD) in developed countries^5–7^. According to the US Renal Data System 2020 Annual Data Report, the incidence rate of dialysis in Taiwan was 523 per million population in 2018^8^. In Taiwan, DKD is also the leading cause of ESRD, accounting for approximately 45% of all people on chronic dialysis in the country. The prevalence of chronic dialysis was 1.91% among patients with type II DM^9,10^. The development of DKD and ESRD may result from a diabetic environment involving glycation end products as well as hemodynamic and hormonal changes due to DM^11^. The risk factors for DKD include older age, male sex, race, a family history of DKD, hypertension, high protein dietary habits, and obesity^12^. Poor glycemic control is an independent predictor of progression to ESRD. Therefore, intensive blood glucose control in the early stages of DM may provide protection against the development of DKD^13,14^.

Early diagnosis of DM is necessary to achieve early blood glucose control. However, the initial symptoms of DM are not obvious, and a large proportion of diabetic patients may have already developed DM-related complications by the time of their diagnosis, such as retinopathy, nephropathy, and neuropathy. In this study, we hypothesized that patients with DM-related comorbidities at the time of their diagnosis, meaning those without early blood glucose control, may have a higher risk of rapid development of ESRD. Thus, we investigated the incidence of dialysis within 5 years among patients with and without DM-related complications at the time of diagnosis. The results of the study could provide a basis for the development of early diabetes screening policies.

## Results

### Demographic and clinical characteristics of the study population

From Taiwan’s National Health Insurance Research Database (NHIRD), we identified 1,081,299 patients with new diagnoses of DM between 2005 and 2013. We then excluded 30,797 patients with a diagnosis of type I DM and 107 patients with gestational DM. Another 7558 patients were excluded because they had a prior history of renal transplant or ESRD. Next, a further 26,958 individuals were excluded because they were younger than 20 years old or had missing data, such as that related to their education level, marital status, urbanization degree of household’s residential area, and prior treatment at major medical institutions. Thus, a total of 1,016,018 patients with newly diagnosed type II DM were included for analysis in this study.

Among these 1,016,018 patients, 257,859 (25.38%) had one or more DM-related complications at the time of DM diagnosis, with a mean Diabetes Complications Severity Index (DCSI) score of 1.69. Among these patients, 81,761 (31.71%) had neuropathy, 65,561 (25.43%) had retinopathy, and 49,417 (19.16%) had nephropathy. The Supplementary Table S1 presents the distribution of DM-related complications at the time of DM diagnosis. A total of 214,567 patients (21.12%) had one DM-related complication, 37,543 (3.7%) had two complications, and 5749 (0.57%) had $\ge$ 3 complications. People who had received a free adult health examination within 3 years had a lower prevalence of DM-related complications at the time of diagnosis. Female patients had a slightly higher rate of DM-related complications (25.92%) than did male patients (24.93%) at the time of DM diagnosis. In addition, older age, lower education level, lower monthly salary; and prior history of malignancy, hypertension, or stroke were associated with a higher prevalence of DM-related complications. Table 1 presents the detailed demographic characteristics of patients with newly diagnosed DM with and without DM-related complications at the time of diagnosis.

**Table 1:** Demographic characteristics of patients with newly diagnosed type 2 diabetes with and without complications at diagnosis.

Variables	Overall		Without complication		With complication		p^a^	DCSI		p^b^
	N	%	n_0_	%	n_1_	%		mean	SD	
All	1,016,018	100.00	758,159	74.62	257,859	25.38		1.69	0.88	
DCSI score										< 0.001
0	758,159	74.62						–	–	
1	128,939	12.69						1.00	0.00	
2	96,690	9.52						2.00	0.00	
3	18,828	1.85						3.00	0.00	
4	10,335	1.02						4.00	0.00	
≧ 5	3067	0.30						5.39	0.66	
DCSI diseases										
No complication	758,159	74.62						–	–	< 0.001
1 complication	214,567	21.12						1.40	0.49	
2 complications	37,543	3.70						2.93	0.71	
≧3 complications	5749	0.57						4.63	0.99	
Sex							< 0.001			< 0.001
Man	554,759	54.60	416,446	75.07	138,313	24.93		1.77	0.92	
Woman	461,259	45.40	341,713	74.08	119,546	25.92		1.61	0.83	
Age							< 0.001			< 0.001
20–44 years	159,225	15.67	132,224	83.04	27,001	16.96		1.47	0.74	
45–54 years	269,546	26.53	212,154	78.71	57,392	21.29		1.54	0.79	
55–64 years	281,081	27.66	211,721	75.32	69,360	24.68		1.62	0.83	
65–74 years	182,828	17.99	127,384	69.67	55,444	30.33		1.75	0.89	
≧75 years	123,338	12.14	74,676	60.55	48,662	39.45		2.04	1.01	
Marital status							< 0.001			< 0.001
Unmarried	84,394	8.31	65,495	77.61	18,899	22.39		1.70	0.90	
Married	721,658	71.03	544,804	75.49	176,854	24.51		1.66	0.86	
Divorced or widowed	209,966	20.67	147,860	70.42	62,106	29.58		1.79	0.93	
Education level							< 0.001			< 0.001
Elementary school or lower	424,425	41.77	297,524	70.10	126,901	29.90		1.78	0.92	
Junior high school	217,982	21.45	166,184	76.24	51,798	23.76		1.63	0.85	
Senior high school	256,971	25.29	201,149	78.28	55,822	21.72		1.60	0.84	
University or higher	116,640	11.48	93,302	79.99	23,338	20.01		1.60	0.82	
Monthly salary (NTD)							< 0.001			< 0.001
≦17,280	242,487	23.87	174,038	71.77	68,449	28.23		1.78	0.93	
17,281–22,800	396,478	39.02	291,477	73.52	105,001	26.48		1.70	0.89	
22,801–28,800	83,050	8.17	63,834	76.86	19,216	23.14		1.61	0.84	
28,801–36,300	87,955	8.66	67,889	77.19	20,066	22.81		1.60	0.82	
36,301–45,800	103,244	10.16	79,920	77.41	23,324	22.59		1.59	0.81	
≧45,801	102,804	10.12	81,001	78.79	21,803	21.21		1.63	0.84	

Variables	Overall		Without complication		With complication			DCSI		p-value
	N	%	n_0_	%	n_1_	%	p^a^	mean	SD	
Urbanization of residence areas							< 0.001			< 0.001
Level 1	209,544	20.62	157,868	75.34	51,676	24.66		1.68	0.87	
Level 2	273,772	26.95	206,590	75.46	67,182	24.54		1.68	0.88	
Level 3	204,312	20.11	154,023	75.39	50,289	24.61		1.69	0.88	
Level 4	174,401	17.17	128,623	73.75	45,778	26.25		1.71	0.89	
Level 5	33,913	3.34	24,244	71.49	9669	28.51		1.76	0.93	
Level 6	60,587	5.96	43,379	71.60	17,208	28.40		1.71	0.90	
Level 7	59,489	5.86	43,432	73.01	16,057	26.99		1.70	0.88	
CCI							< 0.001			< 0.001
0 point	661,975	65.15	532,457	80.43	129,518	19.57		1.48	0.75	
1 point	235,518	23.18	164,762	69.96	70,756	30.04		1.74	0.86	
2 points	73,499	7.23	42,214	57.43	31,285	42.57		1.96	0.93	
≧3 points	45,026	4.43	18,726	41.59	26,300	58.41		2.31	1.08	
Cancer							< 0.001			< 0.001
No	969,281	95.40	724,441	74.74	244,840	25.26		1.69	0.88	
Yes	46,737	4.60	33,718	72.14	13,019	27.86		1.77	0.87	
Hypertension							< 0.001			< 0.001
No	525,623	51.73	412,068	78.40	113,555	21.60		1.56	0.81	
Yes	490,395	48.27	346,091	70.57	144,304	29.43		1.80	0.92	
Stroke							< 0.001			< 0.001
No	976,720	96.13	744,925	76.27	231,795	23.73		1.62	0.85	
Yes	39,298	3.87	13,234	33.68	26,064	66.32		2.34	0.96	
Free adult health exam within 3 years							< 0.001			< 0.001
No	516,713	50.86	389,545	75.39	127,168	24.61		1.74	0.90	
Yes	499,305	49.14	368,614	73.83	130,691	26.17		1.65	0.87	

*SD* standard deviation, *DCSI* diabetes complications severity index, *CCI* Charlson comorbidity index.

^a^chi-square test, ^b^*t* test and ANOVA.

### Bivariate analysis for risk factors of dialysis within 5 years after DM diagnosis

A total of 7185 patients (0.71%) with diabetes needed dialysis within 5 years after their diagnosis. Furthermore, diabetes patients with complications at the time of diagnosis had a significantly higher risk of progressing to requiring dialysis within 5 years compared with those without complications at the time of diagnosis (2.14% versus 0.22%, *P* < 0.001). Next, a higher DCSI score was associated with a higher 5-year cumulative incidence rate of dialysis after diabetes diagnosis (0.38% when the DCSI score was 1; 12.55% when the DCSI score was $\ge$ 5). The number of DM-related complications was also associated with the risk of dialysis within 5 years of diagnosis. Of the patients with one DM-related complication at the time of DM diagnosis, 1.54% required dialysis within 5 years. When two or three or more DM-related complications were present at the time of DM diagnosis, the 5-year cumulative incidence rates of dialysis were 4.67 and 8.12, respectively.

Patients who required dialysis within 5 years of their diagnosis were older than those who did not (59.27 ± 13.87 versus 57.74 ± 13.41, *P* < 0.001). Additionally, the rate of dialysis within 5 years of diabetes diagnosis increased with age group (0.61% in the 20–44 years group versus 0.97% in the ≧75 years group). Male diabetes patients had a slightly higher risk of requiring dialysis within 5 years compared with female patients (0.81% versus 0.58%, *P* < 0.001), and the 5-year cumulative incidence rate of dialysis was higher among older DM patients. In addition, DM patients who were married, had a higher education level, had a higher monthly salary, underwent a free adult health examination within 3 years, joined a pay-for-performance diabetes care program, had fewer comorbidities, had a higher primary physician service volume, and underwent care at a primary clinic or from a nonpublic health-care organization were associated with a lower 5-year incidence rate of dialysis. A detailed comparison of the risk factors for the 5-year cumulative incidence of dialysis is provided in Table 2.

**Table 2:** Comparison of 5-year cumulative incidence rates of dialysis between type 2 diabetes patients with and without complications at diagnosis.

Variables			All				No dialysis			Dialysis within 5 years				p^a^
			N		%		n_0_		%	n_1_		%		
All			1,016,018		100.00		1,008,833		99.29	7185		0.71		
Diabetic complications at diagnosis														< 0.001
No			758,159		74.62		756,502		99.78	1657		0.22		
Yes			257,859		25.38		252,331		97.86	5528		2.14		
DCSI score														< 0.001
0			758,159		74.62		756,502		99.78	1657		0.22		
1			128,939		12.69		128,448		99.62	491		0.38		
2			96,690		9.52		93,788		97.00	2902		3.00		
3			18,828		1.85		18,126		96.27	702		3.73		
4			10,335		1.02		9287		89.86	1048		10.14		
≧5			3067		0.30		2682		87.45	385		12.55		
DCSI complications														< 0.001
No complication			758,159		74.62		756,502		99.78	1657		0.22		
1 complication			214,567		21.12		211,261		98.46	3306		1.54		
2 complications			37,543		3.70		35,788		95.33	1755		4.67		
≧3 complications			5749		0.57		5282		91.88	467		8.12		
Sex														< 0.001
Man			554,759		54.60		550,258		99.19	4501		0.81		
Woman			461,259		45.40		458,575		99.42	2684		0.58		
Age														< 0.001
20–44 years			159,225		15.67		158,248		99.39	977		0.61		
45–54 years			269,546		26.53		267,611		99.28	1935		0.72		
55–64 years			281,081		27.66		279,329		99.38	1752		0.62		
65–74 years			182,828		17.99		181,506		99.28	1322		0.72		
≧75 years			123,338		12.14		122,139		99.03	1199		0.97		
Mean ± SD			57.75 ± 13.41				57.74 ± 13.41			59.27 ± 13.87				
Marital status														< 0.001
Unmarried			84,394		8.31		83,507		98.95	887		1.05		
Married			721,658		71.03		717,070		99.36	4588		0.64		
Divorced or widowed			209,966		20.67		208,256		99.19	1710		0.81		
Education level														< 0.001
Elementary school or lower			424,425		41.77		421,206		99.24	3219		0.76		
Junior high school			217,982		21.45		216,252		99.21	1730		0.79		
Senior high school			256,971		25.29		255,328		99.36	1643		0.64		
University or higher			116,640		11.48		116,047		99.49	593		0.51		
Monthly salary (NTD)														< 0.001
≦17,280			242,487		23.87		240,055		99.00	2432		1.00		
17,281–22,800			396,478		39.02		393,725		99.31	2753		0.69		
22,801–28,800			83,050		8.17		82,564		99.41	486		0.59		
28,801–36,300			87,955		8.66		87,456		99.43	499		0.57		
36,301–45,800			103,244		10.16		102,708		99.48	536		0.52		
≧45,801			102,804		10.12		102,325		99.53	479		0.47		
Urbanization of residence areas														0.479
Level 1			209,544		20.62		208,057		99.29	1487		0.71		
Level 2			273,772		26.95		271,872		99.31	1900		0.69		
Level 3			204,312		20.11		202,807		99.26	1505		0.74		
Level 4			174,401		17.17		173,146		99.28	1255		0.72		
Level 5			33,913		3.34		33,690		99.34	223		0.66		
Level 6			60,587		5.96		60,166		99.31	421		0.69		
Level 7			59,489		5.86		59,095		99.34	394		0.66		
CCI														< 0.001
0 point			661,975		65.15		658,086		99.41	3889		0.59		
1 point			235,518		23.18		234,478		99.56	1040		0.44		
2 points			73,499		7.23		72,358		98.45	1141		1.55		
≧3 points			45,026		4.43		43,911		97.52	1115		2.48		
Cancer														0.010
No			969,281		95.40		962,408		99.29	6873		0.71		
Yes			46,737		4.60		46,425		99.33	312		0.67		
Hypertension														< 0.001
No			525,623		51.73		522,413		99.39	3210		0.61		
Yes			490,395		48.27		486,420		99.19	3975		0.81		
Stroke														< 0.001
No			976,720		96.13		969,953		99.31	6767		0.69		
Yes			39,298		3.87		38,880		98.94	418		1.06		
Pay-for-performance diabetes care program														< 0.001
Not joined			626,040		61.62		620,213		99.07	5827		0.93		
Joined			389,978		38.38		388,620		99.65	1358		0.35		
Free adult health exam within 3 years														< 0.001
No			516,713		50.86		511,974		99.08	4739		0.92		
Yes			499,305		49.14		496,859		99.51	2446		0.49		
Primary physician’s service volume														< 0.001
Low			255,355		25.13		253,022		99.09	2333		0.91		
Moderate			506,779		49.88		502,919		99.24	3860		0.76		
High			253,884		24.99		252,892		99.61	992		0.39		
Level of health care organization														< 0.001
Medical center			198,117		19.50		196,059		98.96	2058		1.04		
Regional hospital			308,905		30.40		306,336		99.17	2569		0.83		
District hospital			174,436		17.17		173,126		99.25	1310		0.75		
Primary clinics			334,560		32.93		333,312		99.63	1248		0.37		
Ownership of health care organization														0.002
Public			222,032		21.85		220,372		99.25	1660		0.75		
Non-public			793,986		78.15		788,461		99.30	5525		0.70		

^a^log-rank test.

### Competing risk models and adjusted cumulative incidence for dialysis

**Figure 1:**
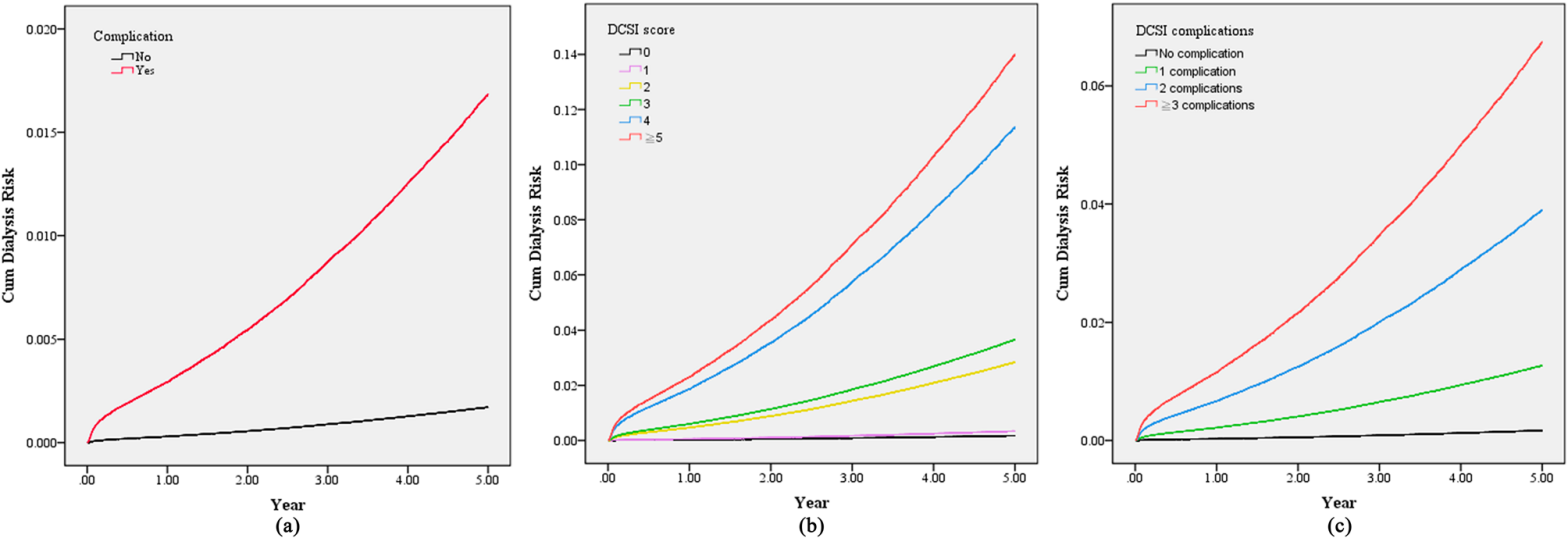
Adjusted 5-year cumulative incidence rates of dialysis after diabetes diagnosis. Adjusted for age, sex, marital status, education level, monthly salary, urbanization of household residential area, Charlson Comorbidity Index score, history of cancer, history of hypertension, history of stroke, enrollment in pay-for-performance diabetes care program, receipt of free adult health examination within 3 years, primary physician’s service volume, primary physician’s level of health-care organization, and primary physician’s ownership of health-care organization. (**a**) shows the adjusted 5-year cumulative incidence rates of dialysis of patients with and without diabetes-related complications at diagnosis. (**b**) shows the adjusted 5-year cumulative incidence rates of patients with varying Diabetes Complications Severity Index (DCSI) scores. (**c**) shows the adjusted 5-year cumulative incidence rates of patients with varying numbers of diabetes-related complications.

Table 3 shows the results of the competing risk models for the risk of requiring dialysis within 5 years among patients with newly diagnosed diabetes. Among 1,016,018 patients with type II DM included in this study, there were 110,004 (10.83%) patients died before dialysis within 5 years of DM diagnosis. As mentioned, the multivariable competing risk models included 15 pre-specific prognostic covariates. Model A demonstrated that the patients with DM-related complications at the time of diabetes diagnosis had a 9.55-times higher risk of dialysis within 5 years compared with those without complications at diagnosis (adjusted hazard ratio [HR]: 9.55, 95% confidence interval CI 9.02–10.11, *P* < 0.001). Model B demonstrated that the risk of dialysis within 5 years significantly increased with the DCSI score. Compared with the patients without DM-related complications, those with DCSI scores of 1, 2, 3, 4, and $\ge$ 5 had 1.97-, 15.33-, 20.42-, 55.05-, and 67.56-times higher risks of dialysis within 5 years, respectively. Model C indicated that the patients with one DM-related complication at the time of diabetes diagnosis had a 7.26-times higher risk of dialysis within 5 years compared with those without DM-related complications (adjusted HR: 7.26, 95% CI 6.83–7.71, *P* < 0.001). The patients with 2 and $\ge$ 3 DM-related complications had even higher risks of dialysis within 5 years, as presented in Table 3 (adjusted HR: 21.66, 95% CI 20.11–23.33, *P* < 0.001 and adjusted HR: 36.12, 95% CI 32.28–40.41, *P* < 0.001, respectively). Figure 1 presents the multivariate-adjusted 5-year cumulative incidence rates of dialysis for diabetes patients with or without DM-related complications, alongside the corresponding DCSI scores, and with 0, 1, 2 or $\ge$ 3 DM-related complications. In addition, we used competing risk models to analyze the risk of dialysis until the end of the observation period in December 2018. A total of 17,348 (1.71%) patients received dialysis until the end of 2018, and 198,646 (19.55%) patients died before dialysis among the included participants. A higher risk of dialysis was observed among patients with DM-related complications at the time of diagnosis compared with those without complications. The risk of requiring dialysis increased with the DCSI scores and the number of DM-related complications. Figure S1 presents the multivariate-adjusted cumulative incidence of dialysis until the end of the observation period in December 2018 (Supplementary Table S2 and Fig. S1).

**Table 3:** Competing risk models for risk of dialysis within 5 years among patients with newly diagnosed diabetes.

Variables	Adjusted model A				Adjusted model B					Adjusted model C			
	HR	95% CI		p-value	HR	95% CI		p-value		HR	95% CI		p-value
With or without diabetes complications at diagnosis													
No (reference)	–	–	–	–									
Yes	9.55	9.02	10.11	< 0.001									
DCSI score													
0 (reference)					–	–	–		–				
1					1.97	1.78	2.17		< 0.001				
2					15.33	14.37	16.36		< 0.001				
3					20.42	18.61	22.42		< 0.001				
4					55.05	50.32	60.22		< 0.001				
≧5					67.56	59.75	76.39		< 0.001				
DCSI complications													
No complication (reference)										–	–	–	–
1 complication										7.26	6.83	7.71	< 0.001
2 complications										21.66	20.11	23.33	< 0.001
≧3 complications										36.12	32.28	40.41	< 0.001
Sex													
Man (reference)	–	–	–	–	–	–	–		–	–	–	–	–
Woman	0.78	0.74	0.82	< 0.001	0.92	0.88	0.97		0.002	0.81	0.77	0.85	< 0.001
Age													
20–44 years(reference)	–	–	–	–	–	–	–		–	–	–	–	–
45–54 years	1.22	1.12	1.32	< 0.001	1.11	1.02	1.21		0.013	1.17	1.08	1.27	< 0.001
55–64 years	0.91	0.84	1.00	0.054	0.79	0.72	0.86		< 0.001	0.86	0.79	0.94	0.001
65–74 years	0.81	0.73	0.90	< 0.001	0.65	0.59	0.73		< 0.001	0.75	0.67	0.83	< 0.001
≧75 years	0.63	0.57	0.70	< 0.001	0.45	0.40	0.50		< 0.001	0.56	0.50	0.62	< 0.001

The above models were adjusted for age, sex, marital status, education level, monthly salary, urbanization of household residential area, Charlson Comorbidity Index score, history of cancer, history of hypertension, history of stroke, enrollment in pay-for-performance diabetes care program, receipt of free adult health examination within 3 years, primary physician’s service volume, primary physician’s level of health-care organization, and primary physician’s ownership of health-care organization.

## Discussion

This study demonstrated that approximately 25.38% of the analyzed patients had DM-related complications at the time of their diagnosis. Those with complications at the time of DM diagnosis had a higher incidence of dialysis within 5 years. Furthermore, compared with those without complications at DM diagnosis, those with complications had a 9.55 times higher risk of requiring dialysis within 5 years. Next, the risk of requiring dialysis within 5 years increased with the DCSI scores and the number of DM-related complications. Patients with a DCSI score of 1 had a 1.97 times higher risk of dialysis than those without DM-related complications. However, when the DCSI score was more than 5, the risk of dialysis within 5 years was 67.56 times higher. Moreover, patients with three or more DM-related complications had a 36.12 times higher risk of dialysis compared with those without any DM-related complications. To the best of our knowledge, this study was the first to compare the risk of rapid progression to ESRD among diabetic patients with and without complications at the time of diagnosis.

The prevalence of undiagnosed diabetes remained high because patients can be asymptomatic until complications develop. According to an international diabetes federation report published in 2019, half of the global diabetic population is unaware of their condition^1^. In Germany, a nationwide sample of 35,869 primary care patients screened in 2005 revealed that the prevalence of type 2 DM was 12.2%, including 23% undiagnosed subjects^15^. Furthermore, the ESTEBAN Survey, conducted in France between 2014 and 2016, revealed that 23% of all diabetes cases were undiagnosed^16^. Next, according to a 2015 national cross-sectional study involving 19,935 respondents conducted in Malaysia, the overall prevalence of type 2 DM was 17.5%; the prevalence of undiagnosed type 2 DM was 9.2%, suggesting that 52% of all individuals with type 2 DM remain undiagnosed^17^. Similar to previous reports, the present study found that 25.38% of the type 2 DM population had complications at the time of diagnosis. Among the analyzed patients, 21.12% had one DM-related complication, and 3.70% had two DM-related complications. Regarding complication severity, 12.69% of the patients had a DCSI score of 1, and 9.52% had a DCSI score of 2. The mean DCSI score for the patients with complications was 1.69 ± 0.88.

Individuals with undiagnosed diabetes are reportedly at a high risk of developing complications if they remain untreated^18^. In addition, diabetes patients with poor glycemic control reportedly have a relatively high risk of developing DM-related complications^19^. Hyperglycemic states can lead to macrovascular complications—such as coronary artery disease, peripheral arterial disease, and stroke—and microvascular complications, including diabetic nephropathy, neuropathy, and retinopathy^20^. The UK Prospective Diabetes Study (UKPDS) revealed that intensive glucose therapy in patients with newly diagnosed type 2 diabetes reduces their risk of developing microvascular complications^13^. Furthermore, a subsequent 10-year follow-up revealed a reduced risk of myocardial infarction and all-cause mortality^21^. Therefore, individuals who are at risk of type 2 diabetes should be screened to initiate patient-centered management and minimize the development and progression of microvascular and macrovascular complications, including the prevention or delay of chronic kidney disease^22,23^.

Next, patients with diabetes reportedly have a 1.75–5-times higher risk of developing chronic kidney disease compared with the general population^24,25^. The incidence of DKD is 2–4% among patients with diabetes, and the median time taken to develop diabetic nephropathy is 94.9 months^26,27^. The UKPDS also reported that 29% of the analyzed patients with newly diagnosed type 2 diabetes developed renal impairment during a median follow-up period of 15 years^28^. The risk factors for DKD include several unmodifiable factors, such as genetics, age, sex, and duration of diabetes, and modifiable factors, including glycemic control, blood pressure, lipids, smoking, chronic low-grade inflammation, advanced glycation end products, and physical inactivity^29^. Early management of these modifiable risk factors may improve the prognosis of DKD in diabetic patients^28^. A study conducted in the United States used the National Health and Nutrition Examination Survey to compare the prevalence rates of chronic kidney disease among people without diabetes, those with prediabetes, those with diagnosed diabetes, and those with undiagnosed diabetes and found the rates of these four groups to be 10.6%, 17.7%, 39.6%, and 41.7%, respectively. These results indicate that the prevalence of chronic kidney disease in patients with undiagnosed diabetes is higher than that in patients with prediabetes and that in diagnosed patients^30^. In the present study, we observed that 19.16% of the patients with DM-related complications at the time of diagnosis had nephropathy. That is, the patients with DM-related complications at the time of diagnosis did not start glycemic control interventions early and thus were at a relatively high risk of requiring dialysis within 5 years.

Diabetes is the leading cause of ESRD globally, accounting for 44% of all new ESRD cases^1,31^. In one study, the annual incidence of ESRD ranged from 0.04 to 1.8%^26^. In Japan, the incidence of ESRD is 4.1/1,000 person-years, and renal function deterioration is associated with older age, poor glycemic control, blood pressure, proteinuria, estimated glomerular filtration rate, history of cardiovascular disease, lifestyle factors (e.g. body mass index, low dietary fiber intake, increased high sodium intake, infrequent exercise), and depressive symptoms^32^. In this study, the risk of dialysis within 5 years after a diabetes diagnosis was higher in the older age group than the younger group. In Taiwan, the incidence rate of dialysis was 523 per million population in 2018^8^. In Finland, a report on 421,429 patients with type 2 diabetes revealed that 1516 of these patients developed ESRD and that the cumulative risk rates of ESRD at 10 and 20 years after diabetes diagnosis were 0.29% and 0.74%, respectively^33^. There were 0.71% of patients who needed dialysis within 5 years after the diagnosis of diabetes in this study, which was higher than previous reports. The higher incidence of ESRD in the Western Pacific region, as previously reported, may account for the higher dialysis rates observed in the current study^34^. In addition, patients without diabetic complications at diagnosis had similar dialysis risks compared to previous reports. On the other hand, a higher risk of dialysis was observed in patients with diabetic complications at diagnosis, which may be another reason for the higher rate of dialysis in this study. In addition, previous studies have reported that patients with DM-related complications, such as cardiovascular disease and peripheral arterial disease, are at a relatively high risk of DKD progression^35,36^. Patients with diabetic retinopathy are also at a relatively high risk of DKD^37,38^. Previous studies in Taiwan comparing dialysis risk among diabetic physicians and nurses with the general diabetes population have revealed a higher risk of dialysis among patients with comorbidities^39,40^. Similarly, the results of the present study suggested that the short-term risk of dialysis (5-year cumulative incidence) was 9.55-times higher in patients with DM-related complications than in those without DM-related complications. The higher the DCSI score and the more DM-related complications present at DM diagnosis, the higher the risk of progression to ESRD within 5 years.

The main strength of the present study was its large number of cases and its inclusion of detailed information regarding confounding variables, including medical and socioeconomic information. In addition, the use of population-based databases enabled us to conduct detailed longitudinal tracking. However, this study had some limitations. First, we did not have access to biochemical data for the study patients. The glycemic control status and eGFR of each study patient was not included in the analysis. However, we included DCSI scores and whether the patients had participated in diabetes pay-for-performance programs in our multivariate analysis, and the effect of DM-related complications on the risk of early progression to ESRD remained significant. Second, because the diagnosis of DM and DM-related complications was based on *International Classification of Diseases, Ninth Revision, Clinical Modification* (*ICD-9-CM*) codes, the possibility of miscoding could not be excluded. In this study, to minimize the risk of bias, we defined the presence of disease by at least three outpatient visits or one hospitalization within 1 year, as in a previous study^41^. Finally, although we included factors related to the environment, monthly salaries, marital status, and health behaviors, the NHIRD does not contain information of family history and lifestyle, such as that related to smoking or exercise. In addition, some diseases and risk factors that can affect kidney function, such as the use of non-steroidal anti-inflammatory drugs, lupus nephritis, renal infarction, and genetic diseases such as polycystic kidney disease, were not included in this study. Although we believe that the impact of these factors on the kidneys has been considered in nephropathy in the DCSI score, the effects of these factors on the progression of DKD were not evaluated in this study.

In conclusion, the risk of requiring dialysis within 5 years was significantly higher among patients with DM-related complications at the time of DM diagnosis compared with those without such complications. Therefore, screening for diabetes and the initiation of glycemic control in the early stages of diabetes are recommended to prevent the rapid progression of DKD.

## Methods

### Study design and data sources

We conducted this retrospective cohort study to assess the 5-year cumulative incidence of dialysis among patients with newly diagnosed DM with and without DM-related complications. Taiwan’s National Health Insurance program was launched in 1995 and covers 99.93% of the country’s residents^42,43^. We obtained data from the NHIRD, part of the Health and Welfare Data Science Center, from 2000 to 2018, and then linked these data with further data obtained from the Taiwan Cancer Registry File, the Cause of Death File from the Ministry of Health and Welfare, the Household Registration File from the Ministry of the Interior, and the Registry of Catastrophic Illness. Patients with catastrophic illnesses, including 30 severe illnesses such as cancer, type I diabetes, and congenital diseases, as well as those with ESRD requiring dialysis, can be registered as catastrophic Illness patients. There were more than 4,473 studies using NHIRD published in past two decades^44^, including the annual reports of diabetes and kidney disease^4,10^. The accuracy of NHIRD had been validated previously^41,45^. In the current study, the definition of disease diagnosis and variables were defined along with previous studies.

### Study participants

**Figure 2:**
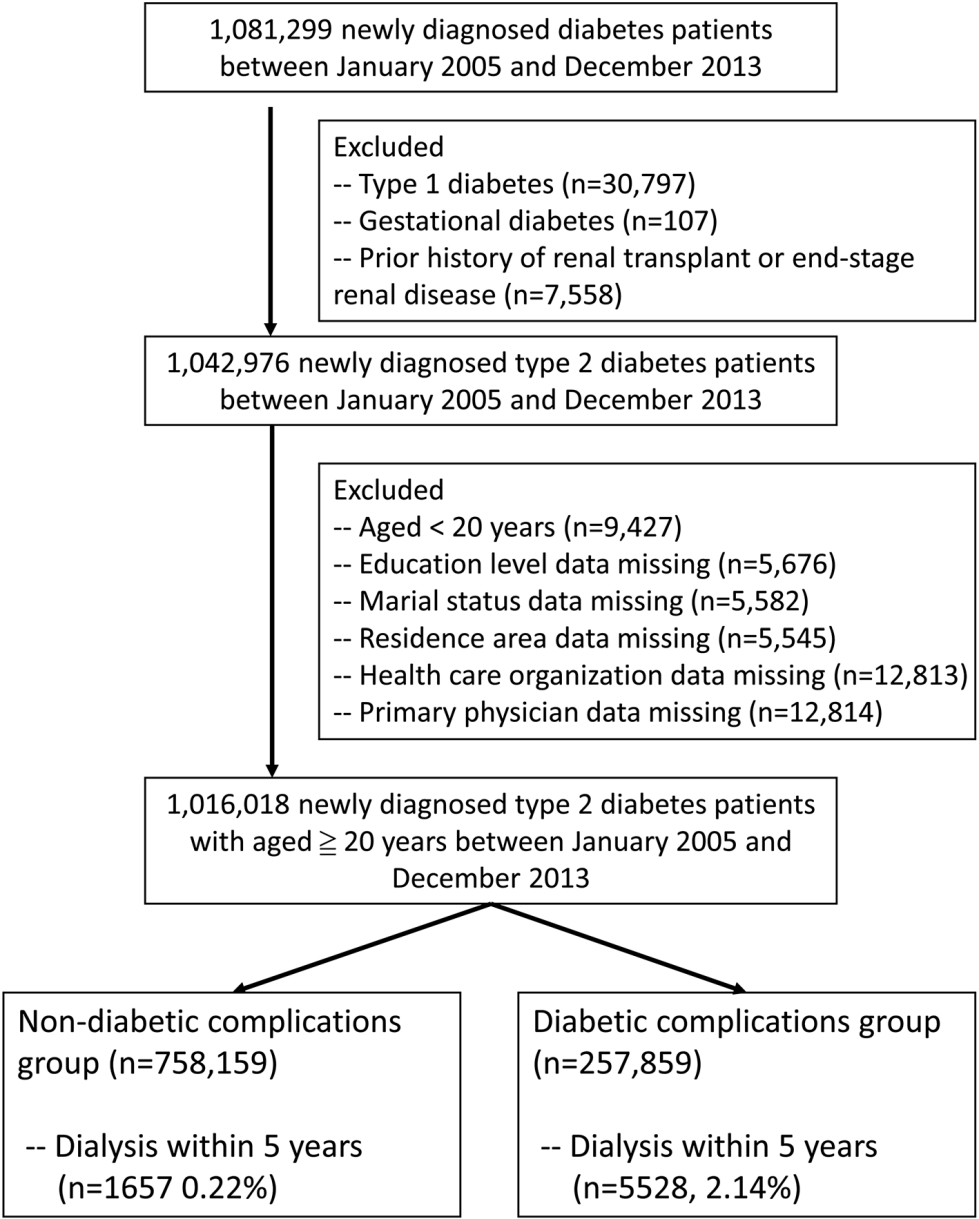
Flowchart of patient enrollment.

Patients with a history of at least one hospitalization and a diagnosis of diabetes (*ICD-9-CM* code 250.x or A181) or a history of three outpatient clinic visits within 365 consecutive days with concurrent use of diabetes medication were defined as diabetic patients^46–48^. Patients older than 20 years with type 2 diabetes newly diagnosed between 2005 and 2013 were also included in this study. Patients were excluded according to the following criteria: (1) A diagnosis of type I DM, which is defined as a catastrophic illness, a history of hospitalization for diabetes ketone acidosis (DKA; *ICD-9-CM* code 250.1x) with outpatient visit diagnosis of insulin-dependent diabetes (ICD-9-CM code 250. × 1 or 250. × 3), or a history of three outpatient clinic visits for insulin-dependent diabetes within 365 consecutive days without DKA hospitalization; (2) those with a diagnosis of gestational diabetes (ICD-9-CM code 648.0 or 648.8); (3) receipt of kidney transplant surgery before DM diagnosis; (4) receipt of renal replacement therapy before DM diagnosis; and (5) incomplete data (Fig. 2).

### Relevant variables

According to the DCSI developed by Young et al. in 2008^49^, DM-related complications can be categorized into seven major categories, namely retinopathy, nephropathy, neuropathy, cerebrovascular disease, cardiovascular disease, peripheral vascular disease, and metabolic disease. In this study, patients who had DM-related complications within 1 year before DM diagnosis were classified as the diabetic complication group, whereas patients without DM-related complications were classified as the nondiabetic complication group. We calculated the DCSI score, a score between 0 and 13 based on the corresponding ICD-9-CM code, of each patient in the diabetic complication group. This group was further divided into five subgroups according to DCSI scores of 1, 2, 3, 4, and $\ge$ 5. We also recorded the number of DM-related complications and further divided the diabetic complication subgroups based on 1, 2, and $\ge$ 3 complications at diagnosis. The distribution of DM-related complications at the time of DM diagnosis was recorded.

To better understand the study participants’ personal characteristics, we retrieved information regarding their age, sex, marital status (unmarried, married, divorced, or widowed), and highest education level (elementary school or lower, junior high school, senior high school, or university or higher) from the database. The economic status of the research participants was divided into six groups according to their monthly salaries: ≤ NT$17,280, NT$17,281–NT$22,800, NT$22,801–NT$28,800, NT$28,801–NT$36,300, NT$36,301–NT$45,800, and ≥ NT$45,801. We also analyzed the urbanization level of residential areas (seven levels, with level 1 being the most urbanized^50^) as an environmental factor. Information of mortality after diabetes diagnosis were obtained from the Cause of Death File from the Ministry of Health and Welfare.

Patients with a history of cancer in the Registry of Catastrophic Illness were considered to have a history of malignancy. Patients were considered to have comorbidities, such as hypertension, stroke and diseases listed in the Charlson Comorbidity Index (CCI)^51^ if they had received a primary or secondary diagnosis based on an *ICD-9-CM* code either twice during outpatient visits or once during hospitalization. CCI scores were calculated for all the participants. To avoid the dual calculation of CCI scores with DCSI scores, diabetes-related diagnoses, stroke-related diagnoses, and cancer-related diagnoses were not included in the calculation of CCI scores. Information about whether diabetes patients had participated in diabetes pay-for-performance care programs (with or without code E4, the specific treatment code for pay-for-performance programs during outpatient visits) was also collected from the database^48^. The diabetes pay-for-performance program launched by the NHI administration in Taiwan provides a financial incentive to facilitate comprehensive assessment and continuous care for patients with diabetes since 2001. According to previous report, the pay-for-performance program increased proportions of patients with HbA1c < 7% (34.5% vs. 32.4%), blood pressure < 130/80 mmHg (37.7% vs. 30.9%), and low-density lipoprotein cholesterol < 100 mg/dL or total cholesterol < 160 mg/dL^52^.We also determined from the database whether individuals had received a free adult health examination (with or without code 21 or 24, the specific codes at outpatient visits) within 3 years prior to their diabetes diagnosis to represent the health belief factor in this study^53^. Finally, this study examined the characteristics of health-care providers, including each primary physician’s service volume (defined as their annual diabetes patient service volume, calculated using the quartile method^47^), health-care organizational level (medical center, regional hospital, district hospital, or primary clinic), and ownership of their health-care organization (public or nonpublic).

### Outcome measurement

The primary endpoint of this study was the determination of whether patients with newly diagnosed DM required dialysis within 5 years. Information regarding dialysis was obtained from the Registry of Catastrophic Illness. Patients with any one of the aforementioned 30 catastrophic diseases, including ESRD requiring dialysis, can apply for a certificate of catastrophic illness. Patients can then be exempted from copayments of medical expenses if their catastrophic illness is acknowledged by a physician and registered in the NHI program. In Taiwan, patients can only apply for catastrophic illness of dialysis after receiving consecutive dialysis for three months. The application form must be completed by a nephrologist, and the certification of catastrophic illness of dialysis will only be issued after rigorous review. The definition of chronic dialysis was consistent with previous studies^54,55^. The time intervals between DM diagnosis and catastrophic illness registration for the patients in this study were recorded to analyze the cumulative incidence of dialysis. Both 5-year and long term (followed until December 31, 2018) cumulative incidence of dialysis were calculated to realize the influence of diabetes complications of risk of dialysis.

### Statistical analysis

All statistical analyses in this study were performed using SAS (version 9.1; SAS Institute, Cary, NC, USA), and a *P* value of < 0.05 was considered statistically significant. The demographic data of patients with newly diagnosed DM were presented alongside descriptive data according to the presence or absence of DM-related complications at the time of DM diagnosis. Categorical characteristics are presented as numbers and percentages, and differences among these characteristics were analyzed using the chi-square test. Continuous variables are presented as means and standard deviations. Finally, differences among the study groups and subgroups were tested using Student’s *t* test for two groups and analyses of variance (ANOVA) for three or more groups.

The follow-up period began at the time of DM diagnosis and continued until the date of dialysis commencement, death, or the end of the observation period (December 31, 2018). The study patients were divided into two groups based on whether they required dialysis within 5 years of DM diagnosis. Crude case numbers and the cumulative incidence of dialysis within 5 years are presented according to the patients’ characteristics. The log-rank test was conducted to examine differences in the 5-year cumulative incidence rates of dialysis among the subgroups.

For the analysis of dialysis risk among DM patients, death was considered a competing event. To further analyze the risk of dialysis within 5 years among patients with and without DM-related complications and with complications of varying severity, multivariate Cox proportional hazards regression models accounting for competing risk were used with adjustment for 15 prespecified risk covariates (i.e. age; sex; marital status; education level; monthly salary; urbanization level of residential area; CCI score; history of cancer; history of hypertension; history of stroke; enrollment in a pay-for-performance diabetes care program; free adult health exam within 3 years; and primary physician’s service volume, health-care organizational level, and ownership of health-care organization). Three competing risk models—namely those for the presence or absence of DM-related complications, DCSI scores, and the number of DM-related complications—were used to calculate the adjusted hazard ratios (HRs) for the risk of dialysis within 5 years after DM diagnosis. After multivariate adjustment, the adjusted HR for dialysis was calculated for each of the three models. Next, we conducted sensitivity tests. The adjusted cumulative incidence of dialysis until December 31, 2018, was plotted. Three competing risk models with adjustment for the same covariates were used to evaluate differences in the risk of dialysis among patients with and without DM-related complications at diagnosis until December 31, 2018.

All data in this study were anonymized, and personal data could not be identified. The informed consent was waived by the Research Ethics Committee of the Taichung Jen-Ai Hospital. The research was conducted in accordance with the 1964 Declaration of Helsinki and amendments and was approved by the institutional review board (IRB) of the Taichung Jen-Ai Hospital (IRB NO. 109-12), Taiwan.

### Supplementary Information

Supplementary Information.

## Data Availability

The datasets for this study can be found in the National Health Insurance Research Database published by the Ministry of Health and Welfare (https://www.mohw.gov.tw/np-108-2.html).
