# Supplementary material for: Risk of rapid progression to dialysis in patients with type 2 diabetes mellitus with and without diabetes-related complications at diagnosis
Source: Sci Rep. 2023 Sep 29;13:16366. doi: 10.1038/s41598-023-43513-z (PMC10541444; doi:10.1038/s41598-023-43513-z)
Supplement: Supplementary file 1 — Supplementary Information. [file 41598_2023_43513_MOESM1_ESM.docx]

**Supplementary Tables and figures**

**Table S1. Distribution of diabetes-related complications among patients with complications at diagnosis**

|  | With complications at diagnosis | Retinopathy | | Nephropathy | | Neuropathy | | Cerebrovascular | | Cardiovascular | | Peripheral  vascular | | Metabolic | |
| --- | --- | --- | --- | --- | --- | --- | --- | --- | --- | --- | --- | --- | --- | --- | --- |
| Variable |  | n_1_ | % | n_2_ | % | n_3_ | % | n_4_ | % | n_5_ | % | n_6_ | % | n_7_ | % |
| Total | 257859 | 65561 | 100.00 | 49417 | 100.00 | 81761 | 100.00 | 42868 | 100.00 | 36402 | 100.00 | 31114 | 100.00 | 391 | 100.00 |
| DCSI score |  |  |  |  |  |  |  |  |  |  |  |  |  |  |  |
| 1 | 128939 | 37639 | 57.41 | 1719 | 3.48 | 60087 | 73.49 | 179 | 0.42 | 14443 | 39.68 | 14872 | 47.80 | 0 | 0.00 |
| 2 | 96690 | 17949 | 27.38 | 31149 | 63.03 | 8443 | 10.33 | 28847 | 67.29 | 12641 | 34.73 | 8494 | 27.30 | 229 | 58.57 |
| 3 | 18828 | 5668 | 8.65 | 7455 | 15.09 | 10117 | 12.37 | 6570 | 15.33 | 4440 | 12.20 | 3995 | 12.84 | 56 | 14.32 |
| 4 | 10335 | 3132 | 4.78 | 6762 | 13.68 | 1594 | 1.95 | 5420 | 12.64 | 3375 | 9.27 | 2392 | 7.69 | 64 | 16.37 |
| ≧5 | 3067 | 1173 | 1.79 | 2332 | 4.72 | 1520 | 1.86 | 1852 | 4.32 | 1503 | 4.13 | 1361 | 4.37 | 42 | 10.74 |
| DCSI diseases |  |  |  |  |  |  |  |  |  |  |  |  |  |  |  |
| 1 complication | 214567 | 50406 | 76.88 | 32243 | 65.25 | 60087 | 73.49 | 28875 | 67.36 | 23706 | 65.12 | 19021 | 61.13 | 229 | 58.57 |
| 2 complications | 37543 | 12669 | 19.32 | 13888 | 28.10 | 17986 | 22.00 | 11327 | 26.42 | 9826 | 26.99 | 9282 | 29.83 | 108 | 27.62 |
| ≧3 complication | 5749 | 2486 | 3.79 | 3286 | 6.65 | 3688 | 4.51 | 2666 | 6.22 | 2870 | 7.88 | 2811 | 9.03 | 54 | 13.81 |

**Table S2. Competing risk models for risk of dialysis after diabetes diagnosis until the end of 2018**

|  |  | Adjusted Model A | | | | Adjusted Model B | | | | Adjusted Model C | | | |
| --- | --- | --- | --- | --- | --- | --- | --- | --- | --- | --- | --- | --- | --- |
| Variables | | HR | 95% CI | | p-value | HR | 95% CI | | p-value | HR | 95% CI | | p-value |
| With or without diabetes complications at diagnosis | |  |  |  |  |  |  |  |  |  |  |  |  |
|  | No (reference) | 1.00 | - | - | - |  |  |  |  |  |  |  |  |
|  | Yes | 4.02 | 3.91 | 4.14 | <0.001 |  |  |  |  |  |  |  |  |
| DCSI score | |  |  |  |  |  |  |  |  |  |  |  |  |
|  | 0 (reference) |  |  |  |  | 1.00 | - | - | - |  |  |  |  |
|  | 1 |  |  |  |  | 1.60 | 1.52 | 1.69 | <0.001 |  |  |  |  |
|  | 2 |  |  |  |  | 6.12 | 5.87 | 6.36 | <0.001 |  |  |  |  |
|  | 3 |  |  |  |  | 8.15 | 7.56 | 8.63 | <0.001 |  |  |  |  |
|  | 4 |  |  |  |  | 14.31 | 13.29 | 15.39 | <0.001 |  |  |  |  |
|  | ≧5 |  |  |  |  | 17.47 | 14.71 | 18.45 | <0.001 |  |  |  |  |
| DCSI complications | |  |  |  |  |  |  |  |  |  |  |  |  |
|  | No complication (reference) |  |  |  |  |  |  |  |  | 1.00 | - | - | - |
|  | 1 complication |  |  |  |  |  |  |  |  | 3.39 | 3.24 | 3.45 | <0.001 |
|  | 2 complications |  |  |  |  |  |  |  |  | 7.48 | 7.17 | 7.86 | <0.001 |
|  | ≧3 complications |  |  |  |  |  |  |  |  | 12.21 | 11.22 | 12.40 | <0.001 |

The models were adjusted for age, sex, marital status, education level, monthly salary, urbanization of household residential area, Charlson Comorbidity Index score, history of cancer, history of hypertension, history of stroke, enrollment in pay-for-performance diabetes care program, receipt of free adult health examination within 3 years, primary physician’s service volume, primary physician’s level of health-care organization, and primary physician’s ownership of health-care organization.


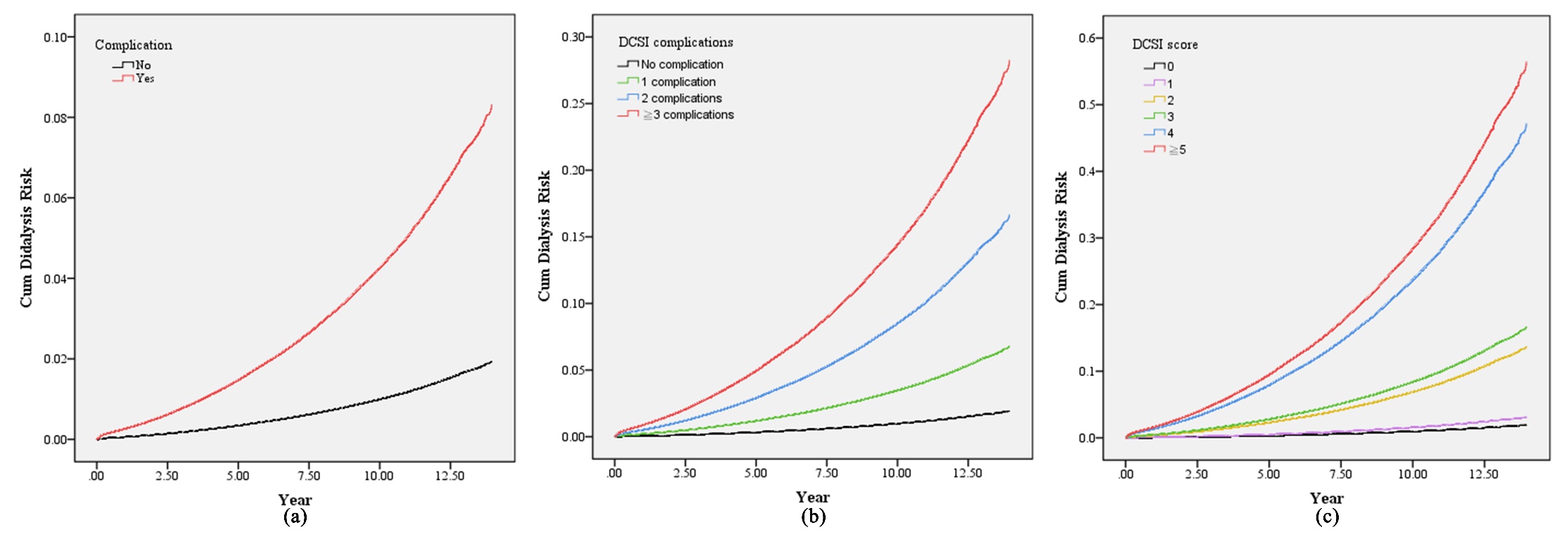
**Figure S1. Adjusted cumulative incidence rates of dialysis after diabetes diagnosis until the end of 2018**

Figure S1 shows the 5-year cumulative incidence of dialysis, which was adjusted for age, sex, marital status, education level, monthly salary, urbanization of household residential area, Charlson Comorbidity Index score, history of cancer, history of hypertension, history of stroke, enrollment in pay-for-performance diabetes care program, receipt of free adult health examination within 3 years, primary physician’s service volume, primary physician’s level of health-care organization, and primary physician’s ownership of health-care organization. Figure S1a shows the adjusted cumulative incidence rates for dialysis in patients with and without diabetes-related complications at diagnosis. Figure S1b shows the adjusted cumulative incidence rates for dialysis in patients with varying Diabetes Complications Severity Index (DCSI) scores. Figure S1c shows the adjusted cumulative incidence rates for dialysis in patients with varying numbers of diabetes-related complications.
